# Supplementary material for: Identification of a Novel Biosurfactant with Antimicrobial Activity Produced by Rhodococcus opacus R7
Source: Microorganisms. 2022 Feb 21;10(2):475. doi: 10.3390/microorganisms10020475 (PMC8877126; doi:10.3390/microorganisms10020475)
Supplement: Supplementary file 1 [file microorganisms-10-00475-s001.zip › microorganisms-1584977-supplementary.pdf]

Table S1. NMR spectra of the biosurfactant compound from *R. opacus* R7 revealing the amino acids composition.

Amino acid	^{1}H	^{13}C
Ala	1.35/3.75	19.88/48.45
Asp	2.9/3.05/3.65	37.05/42.64/53.72
Gly	3.35	40.77
Ser	3.6/3.7	37.05/42.64
Thr	1.2/3.4/3.6	34.13/49.96/53.72

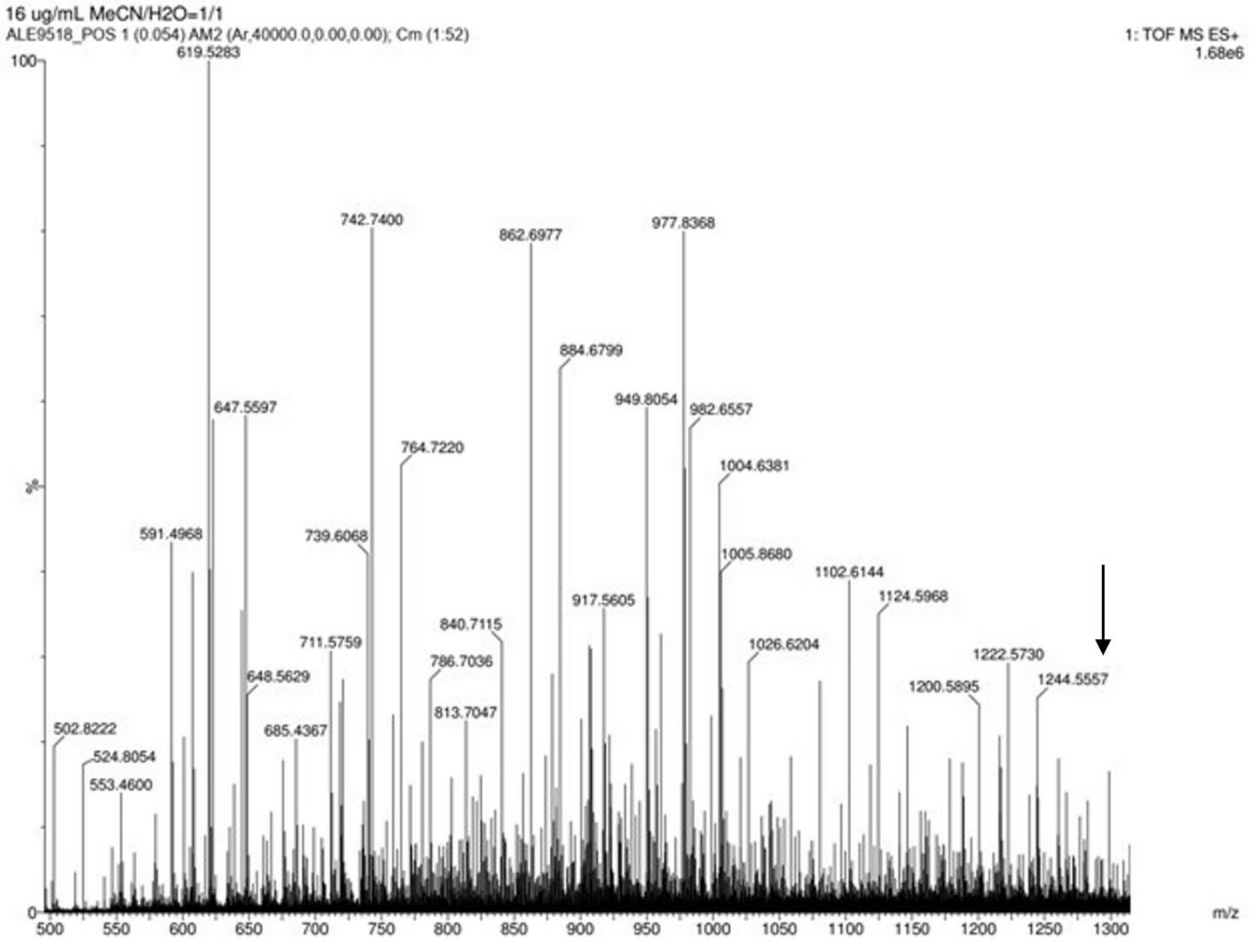

Figure S1. ESI-full mass analysis of the isolated compound. The arrow indicates the mass of 1292 Da.
